# Dietary calcium citrate enhances nutrient digestibility and modulates cecal microbiota function in pre-laying hens

**DOI:** 10.14202/vetworld.2026.821-839

**Published:** 2026-02-28

**Authors:** Elena Yausheva, Tatiana Kholodilina, Elena Sizova, Daniil Shoshin, Kristina Ryazantseva, Ksenia Nechitailo, Tatiana Klimova, Alexandra Mustafina

**Affiliations:** 1 Federal Research Center of Biological Systems and Agrotechnologies of the Russian Academy of Sciences, 29, 9 Yanvarya str., Orenburg, 460000, Russia; 2 Orenburg State University, 13 Prosp. Pobedy, Orenburg, 460018, Russia

**Keywords:** calcium citrate, cecal microbiota, feed efficiency, gut health, nutrient digestibility, poultry nutrition, pre-laying hens, 16S rRNA sequencing

## Abstract

**Background and Aim:**

Calcium source and bioavailability are critical determinants of nutrient utilization, gut microbial ecology, and future productivity in laying hens, particularly during the pre-laying period. Organic calcium salts may exert additional functional effects through microbiota modulation beyond mineral supply alone. This study evaluated the effects of replacing calcium carbonate with calcium citrate on nutrient digestibility, cecal microbiota composition, short-chain fatty acid (SCFA) production, and predicted microbial metabolic pathways in pre-laying hens.

**Materials and Methods:**

Sixty Hisex Brown pre-laying hens (13–20 weeks of age) were allocated to two dietary treatments: a control diet containing calcium carbonate and an experimental diet in which calcium carbonate was fully replaced with calcium citrate. Diets were formulated to be isocaloric, isonitrogenous, and equal in total calcium content. Nutrient digestibility coefficients were determined using a physiological balance trial. Cecal SCFA concentrations were quantified by gas chromatography. Cecal microbiota composition was analyzed by 16S rRNA gene sequencing, and functional pathway prediction was performed using Kyoto Encyclopedia of Genes and Genomes-based bioinformatic analysis. Statistical significance was set at p < 0.05.

**Results:**

Replacement of calcium carbonate with calcium citrate significantly increased the digestibility of crude fat (+28.7%, p ≤ 0.001), crude protein (+7.29%, p ≤ 0.001), calcium (+7.56%, p ≤ 0.05), and phosphorus (+2.92%, p ≤ 0.05). Cecal concentrations of propionic, butyric, and valeric acids were significantly higher in the calcium citrate group (p ≤ 0.001). Microbiota analysis revealed a higher relative abundance of *Bacillota*, particularly *Lactobacillaceae* and *Oscillospiraceae*, and a reduced proportion of Bacteroidota, including *Alistipes*. Alpha diversity indices were higher in the experimental group. Functional prediction indicated enrichment of microbial genes associated with carbohydrate, amino acid (phenylalanine, tyrosine, tryptophan), and fatty acid metabolism, alongside reduced methane metabolism.

**Conclusion:**

Dietary calcium citrate markedly improves nutrient digestibility and beneficially reshapes cecal microbiota composition and function in pre-laying hens. These findings highlight calcium citrate as a promising nutritional strategy to enhance gut health, mineral utilization, and feed efficiency, with potential implications for subsequent egg production and sustainable poultry systems.

## INTRODUCTION

Poultry farming represents a major sector of agriculture and is a key source of high-quality protein for the human population. The ongoing increase in poultry production driven by population growth is closely linked to addressing multiple challenges, particularly those associated with meeting the nutritional requirements of animals [1, 2]. Special emphasis is placed on the pre-laying period in laying hen nutrition, as this phase critically determines subsequent productivity and is accompanied by pronounced physiological changes. During the pre-laying period, substantial alterations occur in protein, lipid, and mineral metabolism to facilitate the accumulation of nutrient reserves required for the forthcoming egg-laying phase. Consequently, the demand for nutrients as well as macro- and microelements increases markedly in laying hens [3].

Among nutritional factors during the pre-laying period, mineral supply, especially calcium, has received considerable attention. Calcium concentration in poultry diets directly affects skeletal integrity and eggshell quality, and calcium deficiency in laying hens contributes to osteoporosis and significant production losses. Nevertheless, extensive evidence indicates that the physiological roles of calcium extend beyond bone development and eggshell formation [4]. Calcium participates in acid–base regulation, supports gastrointestinal enzymatic activity, and interacts closely with the intestinal microbiota, thereby influencing overall digestive processes [5]. Several studies have demonstrated clear associations between calcium source, nutrient absorption, and gut microbiota composition in animals [6–8].

Calcium is supplied to poultry diets in compounds of diverse chemical forms. Calcium carbonate, mainly derived from limestone, remains the most commonly used supplemental calcium source. However, due to its relatively low bioavailability and its impact on gastric pH, limestone-derived calcium can reduce the digestibility of other essential nutrients, including nitrogen and phosphorus [9–11]. With modern poultry production increasingly focused on improving feed efficiency and minimizing environmental pollution, the identification of more bioavailable calcium sources has become a priority. Alternative calcium sources include citrate, lactate, glycerophosphate, gluconate, sulfate, and aspartate. Among these, organic forms such as calcium citrate have attracted growing interest. Calcium citrate offers several advantages, including pH-independent absorption in the gastrointestinal tract, high nutritional value, and superior solubility. A previous study has shown that citrate enhances macronutrient digestion and absorption in laying hens [12]. Although calcium citrate has been reported to improve tibia characteristics in broilers, information on the effects of different calcium forms on the poultry gastrointestinal microbiota remains limited [13].

The poultry gastrointestinal microbiome functions as an active metabolic organ that interacts with the host and plays a crucial role in nutrient utilization. The cecal microbiota, in particular, actively incorporates minerals into its metabolic processes and modulates their availability to the host. Mineral bioavailability influences gut microbiota composition and metabolic activity, which in turn affects feed digestibility and productivity. The cecum harbors the greatest microbial diversity within the poultry gastrointestinal tract, and multiple studies have demonstrated that its activity and taxonomic structure are closely associated with feed efficiency and fat deposition in chickens [14, 15]. The relationship between cecal enzymatic activity and poultry productivity is well documented [16], and cecal microbiota composition has been shown to correlate with calcium deposition in eggshells of laying hens [17].

Despite extensive evidence highlighting the importance of calcium nutrition for skeletal integrity, eggshell quality, and overall productivity in laying hens, most existing studies have primarily focused on inorganic calcium sources and performance outcomes during peak or late laying phases. Comparatively limited attention has been given to the pre-laying period, a critical physiological window characterized by rapid metabolic adaptation and active establishment of the gastrointestinal microbiota. Moreover, although organic calcium sources such as calcium citrate are recognized for their higher bioavailability, their specific effects on nutrient digestibility in relation to cecal microbiota composition, microbial metabolic activity, and short-chain fatty acid (SCFA) production remain insufficiently characterized. In particular, integrative evidence linking calcium source–driven shifts in cecal microbial taxa with functional metabolic pathways and mineral utilization efficiency in laying hens is scarce. This knowledge gap limits the development of targeted nutritional strategies that simultaneously optimize mineral metabolism, gut health, and feed efficiency during early production stages.

The present study aimed to evaluate the effects of replacing calcium carbonate with calcium citrate in the diet of pre-laying hens on nutrient digestibility, cecal microbiota composition, SCFA profiles, and predicted microbial metabolic pathways. Specifically, the study sought to elucidate the relationships between calcium source, mineral and macronutrient utilization, and cecal microbial structure and function, as assessed using 16S rRNA gene sequencing and bioinformatic functional prediction. It was hypothesized that dietary calcium citrate would enhance nutrient digestibility and beneficially modulate cecal microbiota and associated metabolic functions, thereby providing a mechanistic basis for improved feed efficiency and intestinal health in laying hens during the pre-laying period.

## MATERIALS AND METHODS

### Ethical approval

All animal care and experimental procedures were conducted in strict accordance with official guidelines and recommendations. These include the Interparliamentary Assembly Model Laws for Commonwealth of Independent States members (About the treatment of animals, Article 20) and the Guidelines for Working Laboratory Animals of the Federal Research Center for Biological Systems and Agrotechnologies of the Russian Academy of Sciences (FRC BST RAS), 29 9 Yanvarya St., Orenburg, Russia. The experimental methodology was approved by the Ethics Committee of the FRC BST RAS (No. 2 as of 21.03.2024). All procedures comply with the ARRIVE 2.0 guidelines. Humane endpoints were strictly defined and monitored: criteria for intervention included body weight loss exceeding 25% of baseline, refusal of food and water for 48 hours, signs of severe infectious disease or intractable distress (such as depression, dyspnea, or an inability to independently reach resources), and the presence of severe injuries. When necessary, euthanasia was performed in strict accordance with the AVMA Guidelines for the Euthanasia of Animals, 2020 Edition. Animals received visual assessments twice daily throughout the experimental period to ensure their well-being. Every effort was made to minimize animal suffering.

### Study period and location

The study was conducted from May to July 2024 at the FRC BST RAS.

### Experimental design and grouping of the samples

The layers of the Hisex Brown cross were the study object. For the study, 60 heads of replacement young stock of the industrial flock, aged 13 ± 1 weeks, were selected. The laying hens were individually banded and weighed. They were then assigned to weight-matched pairs using the analogous method, ensuring that the live weights within each pair differed by no more than ±30 g. Birds were randomly allocated to individual cages within each resulting pair (n = 30). The study groups were structured with three replicates per group, each containing 10 birds, with every cage considered an independent experimental unit: I (control)–the basal diet (BD), where limestone flour (OOO Inesko, Russia) was used as a source of calcium carbonate; II –the BD and calcium citrate that replaced limestone flour. Limestone flour contained in the BD was replaced with calcium citrate after a two-week preparatory period (acclimatization) from the 13^th^ up to 15^th^ week.

The duration of the experiment was 7 weeks, from the 13^th^ to the 20^th^ week (adaptation + experimental period). The animals were housed in standard battery cages located on one level, meeting all density standards (not less than 0.09 m^2^/head); the cages were equipped with a trough feeder and a nipple drinker. The duration of lighting was 10 h/day, illumination was 10 lux, and the lighting mode was cyclic (10L:14D) and set using an auto-timer (lights on from 8 a.m. to 12 a.m., 1 p.m. to 5 p.m., 2 a.m. to 4 a.m.). The parameters of the microclimate of the room were controlled and met the requirements [18]. The air temperature was 22°C ± 2°C, humidity 65% ± 5%, and the ventilation system provided an air velocity of 0.8 m/s. The ammonia content in the room air was within acceptable limits, up to 3.12 ± 0.3 mg/m^3^. The hens’ cages were cleaned daily. No recorded deaths occurred during the study period. Daily feed intake was monitored, and individual body weights were assessed weekly. Laying hens were fed once a day at 9:00 a.m.

**Figure 1:**
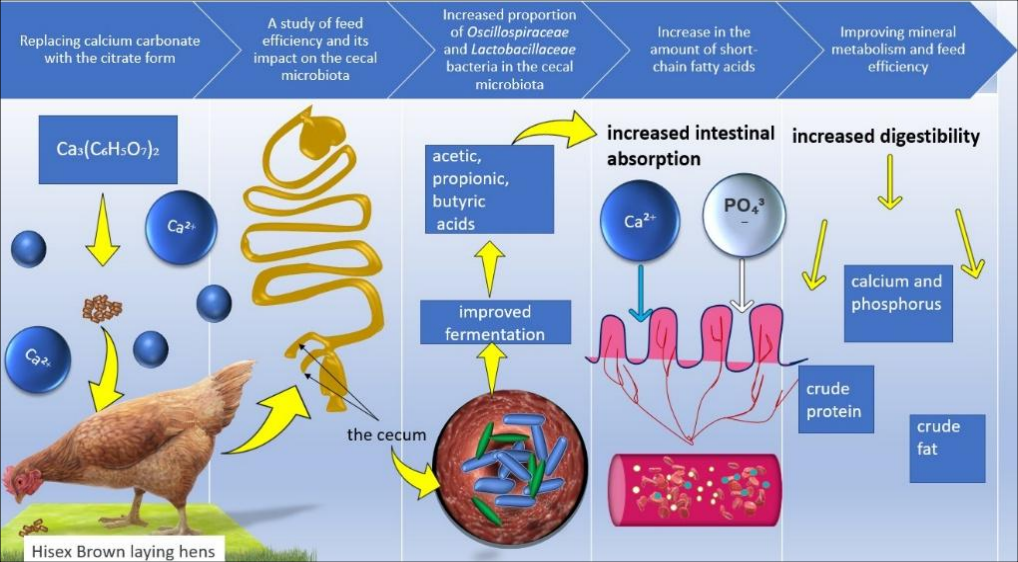
The mechanism of calcium citrate action on the metabolism of laying hens.

A schematic overview of the experimental design, dietary replacement strategy, sampling points, and analytical workflow is presented in Figure 1.

### Diet formulation

Feeding protocols were aligned with age-specific nutritional requirements, following the recommendations of the Federal State Budget Scientific Institution Federal Scientific Center «All-Russian Research and Technological Poultry Institute» [19]. Water was provided *ad libitum* (freely available) to all birds. The diets used in our study were developed to be isocaloric and isonitrogenous (energy and protein matched). The experimental supplement was incorporated by replacing an equivalent amount of nutritionally inert limestone flour, thus maintaining consistent levels of metabolizable energy and crude protein across all diets. The total calcium content remained identical in all groups (Table 1).

**Table 1:** Chemical composition of feed (laboratory data).

Indicator	Control	Group II	p-value
Ca (mg/kg)	24430.52 ± 195.07	24409.90 ± 141.41	0.935
P (mg/kg)	5780.24 ± 67.30	5827.69 ± 93.09	0.662
Crude protein (%)	15.94 ± 0.32	16.21 ± 0.19	0.506
Metabolizable energy (MJ/kg)	11.28 ± 0.05	11.39 ± 0.07	0.862

Subsequent chemical analysis of the finished feeds confirmed no significant differences in the crude protein content between the treatment groups (p ≤ 0.05).

All diets were estimated to contain 11.3 MJ/kg of energy and 16% crude protein. Table 2 shows the main diet’s exact composition. Components were mixed just before feeding using a stepwise method, with calcium content calculated per kilogram of feed. The birds were given the mash feed.

**Table 2:** Composition of laying hens’ basal diet.

Ingredients	Mass of the substance (g/kg)
Wheat	500.00
Barley	200.00
Corn	20.00
Sunflower cake	50.00
Soybean meal	50.00
Fish meal	50.00
Sunflower oil	10.00
Feed yeast	5.00
Bran	40.00
Limestone flour	65.00
Premix	10.00
Total	1000.00

Nutrient content (per 100 g of the basal diet, equivalent to per kg): Metabolizable (exchange) energy: 11.30 MJ/kg, Crude protein: 16.00%, Crude fat: 4.50%, Crude fiber: 4.50%, Starch: 28%, Lysine: 0.71%, Methionine: 0.37%, Methionine + cystine: 0.58%, Calcium: 2.40% (24,000 mg/kg), Total phosphorus: 0.60% (6,000 mg/kg), Digestible phosphorus: 0.51% (likely non-phytate or available P).

### Physiological experiment

Determination of quantitative aspects of metabolism was performed in a physiological experiment. The physiological experiment comprised two periods. The first period was preliminary and lasted for 7 days. The second period (accounting) lasted 5 days. During the accounting period, the amount of consumed feed (according to feed remains in trays), water, and droppings was recorded daily. During the 5-day recording period, feces from each cage were collected twice daily (at 8:00 AM and 4:00 PM) to minimize nitrogen loss. Samples from each cage were pooled to create a single average sample, and a 10% aliquot of the total fecal mass was selected for analysis (n = 3). The collected feces were immediately frozen at –20°C. Subsequently, the samples were dried in an oven at 60°C until a constant mass was achieved, ground, and analyzed for their nutrient, calcium (Ca), and phosphorus (P) contents.

The following formula was used to determine the coefficients for nutrient digestibility: Nutrient digestibility coefficient = [(A – B) / A] × 100%, where: A is the amount of nutrient consumed with feed during the recording period and B is the amount of nutrient excreted in feces during the same period.

### Preparation of calcium citrate

The calcium citrate preparation was obtained from dolomite flour (OOO Akkermancement, Russia). Dolomite flour was added to a solution of technical citric acid (concentration of about 10%) to obtain calcium citrate, and the solution was left in the light for 2 h until foaming ceased. Calcium citrate microcrystals were formed, which were the crystallization centers. The target product (calcium citrate) gradually precipitated. This method does not require a heating system. Furthermore, if it is necessary to obtain salt with the required hydration, a drying cabinet can be used (at a temperature of 80°C – 90°C, 4-hydrate hydration can be achieved in 3-4 h), or air-drying can be performed. The synthesis of calcium citrate tetrahydrate (calcium citrate) was carried out according to the following equation:

3 CaCO_3_ + 2 C_6_H_8_O_7_ + H_2_O = Ca_3_(C_6_H_5_O_7_)_2_ × 4H_2_O + H_2_O + 3 CO_2_

The practical yield of the reaction = 88.77%, which, together with the low production costs, is an advantage of using this method. The hydrodynamic diameter of the calcium citrate particles was determined using DLS with a Microtrac NANOTRAC WAVE II (Microtrac LLC, Moscow, Russia). The resulting average diameter was measured to be 5.9 μm (±0.4 μm).

### Determination of the chemical and elemental compositions

The chemical composition of the droppings and feed samples was studied using standard methods in the Testing Center of the Center for Collective Use of the Federal Scientific Center of Biodiversity of the Russian Academy of Sciences http://цкп-бCT.pф (Orenburg, Russia). The chemical composition of the manure and feed samples was determined using the standard AOAC method (n = 3 replicates per analysis). Crude protein was analyzed via the Kjeldahl method (AOAC 984.13) using a UDK 139 semi-automatic Kjeldahl distillation unit (Velp, Usmate Velate, Italy). Crude fat was determined using the petroleum ether extraction method (AOAC 920.39) with a SER 148/6 device (Velp). Crude fiber was measured using the Weende method (AOAC 962.09) and FIWE fiber analyzers (Velp). A standard sample of Velp Scientific oatmeal (Code A00000318) was included in each batch run for quality control. The crude ash content was determined by dry ashing at 550°C (AOAC 942.05) in a Nabertherm muffle furnace (Nabertherm GmbH, Lilienthal, Germany).

A coefficient of variation (CV) value of < 5% indicated acceptable precision, and recovery rates between 95% and 105% were considered satisfactory. The organic matter content was calculated as the loss of mass during ashing. The nitrogen-free extract was calculated by subtracting the values of crude protein, crude fat, crude fiber, and ash from the dry matter. The elemental composition of the feed and droppings was studied using mass spectrometry on an Agilent 7900 ICP-MS inductively coupled plasma mass spectrometer (Agilent Technologies, Santa Clara, CA, USA). The thermal-oxidative degradation of the organic matrix was performed using a PREEKEM TOPEX+ microwave sample preparation system (PreeKem Scientific Instruments, Shanghai, China). Standard solutions were prepared from a multicomponent mixture procured from Merck (Darmstadt, Germany). For quality control, blank samples were incorporated into each determination; the CV did not exceed 5%.

### Determination of SCFA content

The total amount of SCFA in the cecum was determined using a Crystal LUX 5000 chromatograph (Chromatec, Yoshkar-Ola, Russia) (n = 3). Nitrogen was used as the carrier gas. The detector temperature was 220°C, the injector temperature was 140°C, the hydrogen flow rate was 50 mL/min, the air flow rate was 500 mL/min, and the gas flow rate was 50 ml/min. The flow split was 1:10. The analysis time was 20 min. The isotherm was at 140°C. The concentration is expressed in μmol/g. The detection limit of the device was 0.5 mg/dm^3^. HP-FFAP column, 50 m x 0.32 mm x 0.5 μm. Detector: flame ionization detector. The following comparison samples (Ecotech, Russia) were used (pure substances for chromatography) to calibrate the device: butyric acid (Cas No. 107-92-6, 99.5%), propionic acid (Cas No. 79-09-4, 99.8%), valerianic acid (TU 6-09-528-75, 99.0%), caproic acid (Cas No. 142-62-1, 99.3%), and glacial acetic acid (GOST 61, 99.8%).

### Microbiota analysis

To analyze the gastrointestinal tract microbiota, cecal contents were collected using sterile instruments from three animals in each group. The samples were immediately placed into test tubes containing a preservative solution (DNA/RNA Shield, Zymo Research, Irvine, CA, USA) and frozen (to –80°C) no later than 24 h after collection. Cecal samples for microbiota analysis were collected 6-8 hours after feeding. Samples of large intestinal contents were transported in a cooler with cooling elements (temperature range: +2°C to +6°C). NGS sequencing of the samples was performed no later than 2 months after collection. DNA libraries were prepared, sequencing was performed, and bioinformatics processing was performed at the Center for Collective Use of Scientific Equipment “Persistence of Microorganisms” of the Institute of Cellular and Intracellular Symbiosis, Ural Branch of the Russian Academy of Sciences (Orenburg, Russia).

### DNA extraction and 16S rRNA sequencing

The DNA library was prepared for high-throughput sequencing following the standard Illumina protocol (Part #15044223, Rev. B). Amplicons targeting the V3-V4 hypervariable region of the 16S rRNA gene were generated using the specific primer set S-D-Bact-0341-b-S-17 and S-D-Bact-0785-a-A-21 [20]. Total DNA was isolated from rumen content samples using the Fast DNA® SPIN Kit for Feces (MP Biomedicals Inc., Irvine, CA, USA) using the Lysing Matrix E lining matrix.

DNA purity and concentration were monitored by photometry on a NanoDrop 8000 instrument (Thermo Fisher Scientific Inc., Waltham, MA, USA) and a Qubit 4 fluorometer (Life Technologies, Carlsbad, CA, USA) with a high-sensitivity dsDNA analysis kit (Life Technologies). DNA libraries were purified using Agencourt AMPure XP beads (Beckman Coulter, Brea, CA, USA) and screened by capillary electrophoresis using the QIAxcel DNA kit cartridge (Qiagen, Hilden, Germany) in an advanced Qiaxcel system (Qiagen). Sequencing was performed on the MiSeq platform (Illumina, San Diego, CA, USA) using the MiSeqReagent Kit V3 2×300 (Illumina). Ultrapure nuclease-free water served as a negative control sample for sequencing. ZymoBIOMICS Microbial Community DNA Standard (Zymo Research) served as a positive control sample for sequencing.

### Bioinformatic processing

In the first step, the raw reads obtained from sequencing were assessed using Fast QC (version 0.11.9 [https://www.bioinformatics.babraham.ac.uk/projects/fastqc/]). The assessment was necessary to determine the parameters for further processing and included an assessment of the quality and length of reads and the presence of adapter sequences. Paired reads were merged using USEARCH V 11.0.667 (drive5.com/usearch) using the -fastq_mergepairs command with -fastq_maxdiffs 10 and -fastq_pctid 80. Adapter cutting was performed using cutadapt 1.9.1 [21]. After merging and removing the adapters, the reads were re-evaluated using FastQC v. 0.11.7. Subsequent processing of the merged reads was performed using Usearch V 11.0.667/. The combined reed filtering was carried out according to the following criteria: -fastq_filter: -minlen 380 (bp), -maxee 1.0 (the maximum expected read error is no more than 1 in 100 nucleotides), and the minimum sample length is 400 bp. The filtering quality was assessed using FastQC v 0.11.7. The next step included dereplication (parameter: -fastx_uniques) and clustering of the filtered reads using the UPARSE algorithm were performed [22]. Dereplication and clustering resulted in the formation of operational taxonomic units (OTUs). During the clustering step, chimeric sequences were detected and removed using the UCHIME2 algorithm. The final OTUs were aligned with the original concatenated reads at a similarity level of 97% using a global alignment (usearch_global tool). The number of concatenated reads corresponding to each OTU was estimated as a result of the global alignment. Contaminating OTUs were identified and removed using the usearch_global parameter: -strand plus -id 0.97 by comparing the sequences of the test and negative control samples. The taxonomic classification of the sequences was performed using the SILVA reference database.

All methods were performed in accordance with relevant guidelines and regulations to ensure the reproducibility and comparability of the results.

### Statistical analysis

Statistical analysis was performed to ensure the study results’ reliability and validity. All collected data were analyzed using Statistica 10 (StatSoft, Tulsa, OK, USA) and Microsoft Excel 16 (Microsoft, Redmond, WA, USA) data analysis packages. Descriptive statistics, including mean and standard deviation, were calculated for each parameter to summarize the data. The normality of data distribution was tested using the Shapiro-Wilk test, and the Duncan test was used for the post hoc test. The Mann–Whitney U test was used to compare the differences between the control and experimental groups. Results were considered significant at p < 0.05. Alpha diversity metrics (Chao1, Shannon, and Simpson diversity indices) were calculated to assess the richness, evenness, and overall diversity of microbial communities. Statistical significance was tested using analysis of variance to identify differences in diversity indices between groups. Beta diversity analysis was performed to assess differences in microbial community composition between groups using nonmetric multidimensional scaling based on the Bray–Curtis dissimilarity index. The statistical significance of differences in beta diversity was assessed using permutation multivariate analysis of variance. Correlation analysis was performed to examine the relationships between microbial taxa and ND parameters. Spearman’s rank correlation coefficient was used to identify significant associations given its suitability for nonlinear relationships and ordinal data. OTUs were filtered and assigned taxonomic affiliations for subsequent analyses. Functional profiling of microbial communities was performed using the KEGG database. Statistical comparisons of predicted metabolic pathways were made using MicrobiomeAnalyst (module Marker Data Profiling [Tax4Fun]) and appropriate association tests identifying significant changes in pathway abundance with p-values adjusted using false discovery rate correction to control for multiple comparisons.

## RESULTS

### Nutrient digestibility

**Figure 2:**
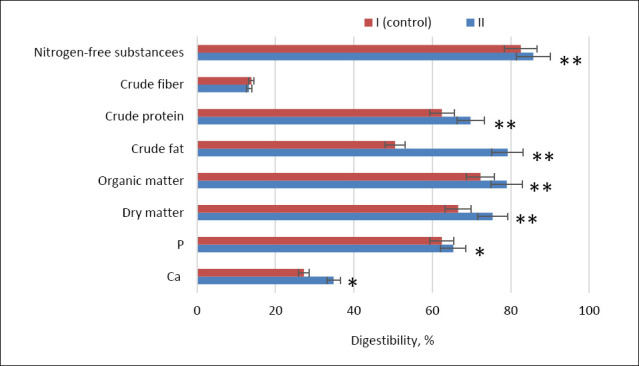
Apparent digestibility coefficients (%) of key dietary nutrients in control (Group I) and experimental (Group II) laying hens. Note: Asterisks indicate significant differences compared to the control: *p ≤ 0.05, **p ≤ 0.001. Ca = calcium; P = phosphorus.

Nutrient digestibility coefficients were calculated. The evaluation of nutrient absorption demonstrated a significant increase in the digestibility of dry matter, organic matter, crude protein, nitrogen-free extractive substances (NFS), and crude fat in the second experimental group compared with the control. The respective increases were 8.8%, 6.7%, 7.29%, 3.21%, and 28.7% (p ≤ 0.001) (Figure 2).

Assessment of mineral assimilation further revealed higher digestibility coefficients of calcium and phosphorus in group II compared with the control by 7.59% and 2.92%, respectively (p ≤ 0.05).

### SCFA profile in the cecum

**Figure 3:**
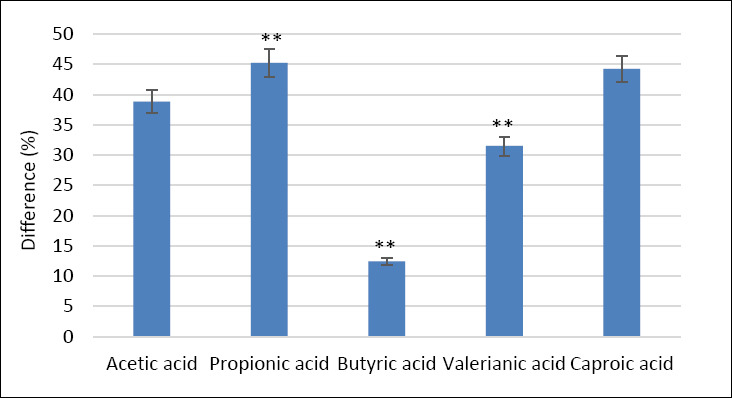
Percentage difference in cecal short-chain fatty acid concentrations in laying hens fed calcium citrate (Group II) compared to the control group (Group I). Bars represent mean differences ± SEM. **p ≤ 0.001 vs. control.

Analysis of SCFA content in the cecum of laying hens showed that group II had significantly higher concentrations of propionic acid (45.2%), butyric acid (12.4%), and valerianic acid (31.5%) compared with the control group (p ≤ 0.001 for all comparisons) (Figure 3).

### Cecal microbiome community structure

**Figure 4:**
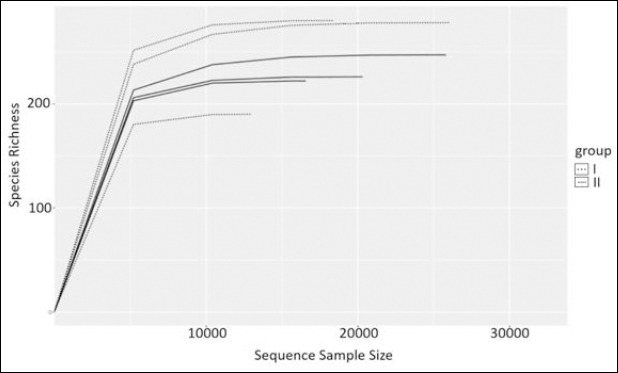
Rarefaction curves (sequence-based resolution) of cecal microbiota samples from control (Group I) and experimental (Group II) laying hens, showing observed species richness as a function of sequencing depth.

Analysis of the cecal microbiota revealed a high diversity of taxonomic groups. Sequencing generated 169,834 reads, ranging from 20,817 to 35,590 raw reads per sample. After merging and filtering, 121,421 reads were retained for downstream analysis. Clustering and removal of singletons and doublets resulted in the identification of 537 OTUs. Rarefaction (resolution) curves constructed from the obtained sequences and OTUs approached a plateau for all samples, indicating sufficient sequencing depth for reliable characterization of the cecal microbiota (Figure 4).

**Figure 5:**
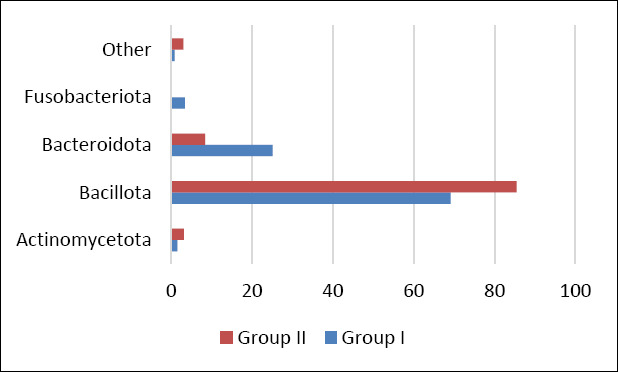
Relative abundance (%) of major bacterial phyla in the cecal microbiome of control (Group I) and experimental (Group II) laying hens. “Other” includes minor taxa each representing ≤2% of the total bacterial community.

Comparative analysis of bacterial communities between control and experimental groups revealed differences in the relative abundance of dominant taxa. In group II, a higher proportion of bacteria belonging to the phylum *Bacillota* and a lower relative abundance of *Bacteroidota* and *Fusobacteriota* were observed compared with the control (Figure 5).

**Figure 6:**
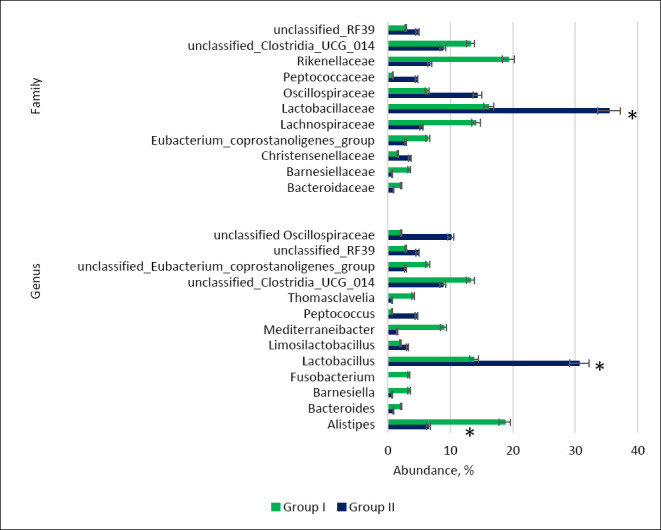
Relative abundance (%) of key bacterial families and genera in the cecal microbiome of control (Group I) and experimental (Group II) laying hens. Asterisks indicate significant differences: *p ≤ 0.05 vs. control.

Replacement of limestone flour with calcium citrate increased the relative abundance of *Lactobacillus*, unclassified *Oscillospiraceae*, and *Oscillospiraceae*, while reducing the representation of *Lachnospiraceae*, *Alistipes*, unclassified *Eubacterium coprostanoligenes*, unclassified Clostridia UCG-014, *Mediterraneibacter*, and *Fusobacterium* (Figure 6).

### Microbial diversity and beta diversity

Microbial diversity analysis demonstrated higher Chao1 richness values in the experimental group than in the control (Table 3). Similarly, Simpson and Shannon diversity indices were higher in group II.

**Table 3:** Indices of species diversity in the ruminal microbiota of bulls after silicon treatment.

Indicator	Control	Group II	p-value
Chao1	284.00 ± 3.21	326.70 ± 1.45	0.04
Simpson	0.83 ± 0.05	0.85 ± 0.04	0.21
Shannon	3.56 ± 0.25	3.66 ± 0.18	0.48

**Figure 7:**
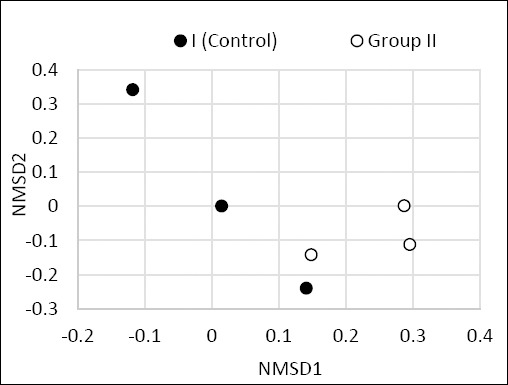
Relative abundance (%) of predominant bacterial families and genera in the cecal microbiome of control (Group I) and experimental (Group II) laying hens.

Beta diversity analysis showed no major differences in the overall organization of bacterial communities between the control and experimental groups (p = 0.045) (Figure 7).

### Correlation between microbiota and nutrient utilization

**Figure 8:**
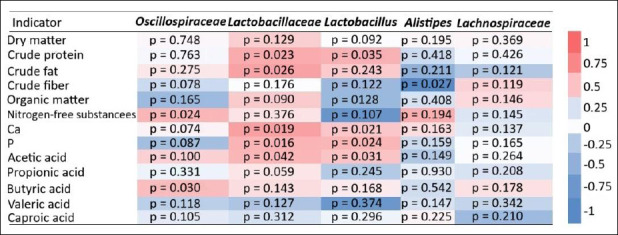
Heatmap of Spearman’s rank correlation coefficients between nutrient digestibility coefficients, cecal short-chain fatty acid concentrations, and taxonomic profiles (at family/genus levels) in the cecal microbiota of laying hens in the experimental group (calcium citrate treatment).

Pearson correlation analysis revealed a positive association between the *Lactobacillaceae* taxon and conversion coefficients of crude fat, crude protein, phosphorus, and calcium (Figure 8). A moderate positive correlation between *Lactobacillaceae* and acetic acid concentration in the cecum was also observed.

Similarly, the *Lactobacillus* taxon showed positive correlations with crude protein, calcium, and phosphorus conversion coefficients. In contrast, a negative correlation was detected between the *Alistipes* taxon and crude fiber digestibility. A moderate positive correlation was found between the *Oscillospiraceae* taxon and the NFS conversion coefficient, as well as between *Oscillospiraceae* and BA.

### Predicted metabolic pathways of the cecal microbiota

**Figure 9:**
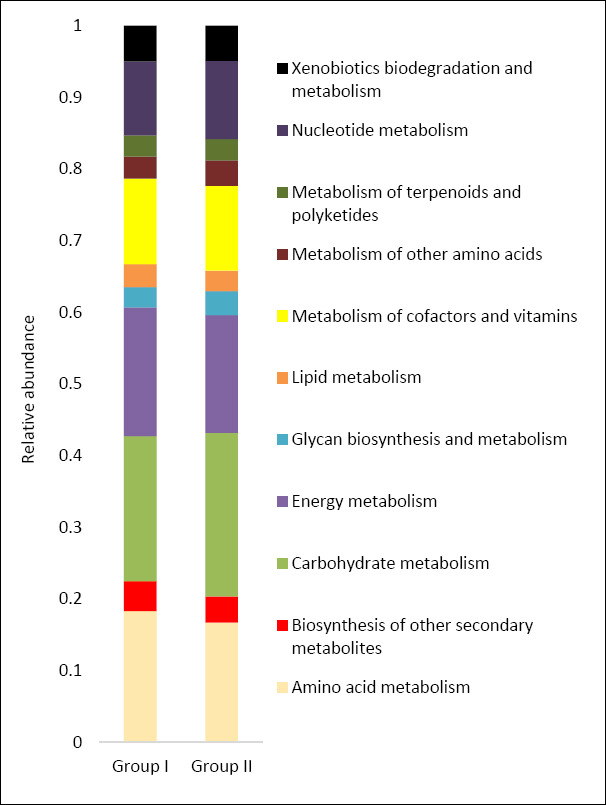
Predicted functional metabolic pathways (based on 16S rRNA or metagenomic inference) of cecal bacteria in control (Group I) and experimental (Group II) laying hens.

Functional profiling based on *16S rRNA* sequences enabled prediction of the metabolic potential of the cecal microbiota in both dietary groups (Figure 9). In both groups, the predominant predicted pathways were associated with carbohydrate, amino acid, cofactor, nucleotide, and energy metabolism.

**Figure 10:**
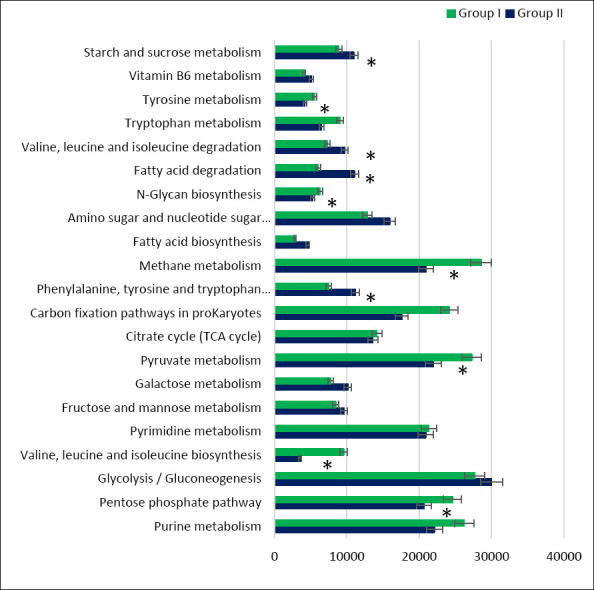
Number of microbial phenotypes associated with predicted metabolic pathways in the cecal microbiome of control (Group I) and experimental (Group II) laying hens. Note: Asterisks indicate significant differences: **p ≤ 0.001 vs. control.

Detailed pathway analysis indicated a higher abundance of genes related to carbohydrate, lipid, and amino acid metabolism in group II compared with the control (Figure 10). Overall, nine predicted microbial pathways differed significantly in gene abundance between diets containing calcium carbonate and calcium citrate.

Compared with the control, the cecal microbiota of group II was enriched in genes associated with starch and sucrose metabolism (p = 1.626e-05), pyruvate metabolism (p = 1.419e-05), and the tricarboxylic acid cycle. Conversely, within carbohydrate metabolism, reduced gene abundance was observed for the pentose phosphate pathway (p = 1.121e-05) and N-glycan biosynthesis (p = 2.224e-05).

Predicted pathways related to amino acid metabolism showed increased gene abundance for phenylalanine, tyrosine, and tryptophan biosynthesis, as well as valine, leucine, and tryptophan biosynthesis (p = 2.781e-05). Analysis of lipid metabolism pathways revealed higher representation of genes associated with fatty acid biosynthesis (p = 1.381e-05) and fatty acid degradation (p = 2.832e-05). In contrast, genes involved in methane metabolism were less abundant in the cecal microbiota of the experimental group than in the control (p = 3.754e-05).

## DISCUSSION

### Role of calcium in poultry nutrition

Calcium is an important macronutrient in poultry. It plays a key role in the development of bone and other systems in the animal body. The use of alternative forms is one of the main areas in poultry farming associated with meeting the calcium needs of farm animals. The widespread use of alternative calcium compounds in poultry farming, including in the form of organic acids, is justified by their greater bioavailability compared to traditional drugs [23]. However, the use of various calcium compounds in the diet of poultry has a different effect on their physiological processes, growth, and productivity, which is due to the level of enzymatic processes in the intestine [24, 25].

### Role of intestinal microbiota in mineral utilization

The intestinal microbiota of farm poultry plays a key role in digestive processes and has a significant impact on the degradation of feed components. The gut microbiota of farm animals actively interact with minerals and uses them in metabolic pathways. The microbiota of the cecum in the gastrointestinal tract of laying hens is the most studied area. The use of active representatives of microbiota in the cecum as biomarkers of feed efficiency in poultry seems prospective [26].

### Knowledge gap during the pre-laying period

Most prior research on calcium supplementation has focused on hens during the peak egg production. In contrast, the pre-laying period, a critical window for intestinal maturation and the foundation of future eggshell formation, has received minimal attention in the scientific literature [27, 28].

### Study contribution and main findings

Our study addressed this oversight by examining the characteristics of nutrient absorption during this key developmental phase. In this study, we demonstrated that including an organic form of calcium (calcium citrate) in the diet enhanced the overall feed efficiency (including calcium utilization) and positively modulated the cecal microbiota.

### Bioavailability advantages of calcium citrate

Calcium citrate has greater bioavailability than inorganic forms of the mineral and does not affect intestinal pH [12]. Calcium citrate is characterized by its highest solubility compared to other organic forms [29].

### Effects of calcium citrate on microbiota diversity

Compared with calcium carbonate, the use of calcium citrate in the diet of laying hens contributed to a greater richness and diversity of the cecal microbiota. This result is consistent with the results of Wang K *et al*. [30], which showed that the use of calcium citrate in the diet increased the microbiota’s richness, uniformity, and diversity and positively affected intestinal health. We assume that the increase in the diversity indices of the cecum microbiota of laying hens in our study could be one of the reasons for the increase in feed efficiency in the experimental groups compared to the control. A study on laying hens and chickens showed a relationship between the improvement of intestinal microbiota diversity and productivity indicators [31, 32].

### Shifts in dominant microbial phyla

In the microbiota of the cecum of laying hens, a higher proportion of bacteria of the *Bacillota* taxon, which are active producers of SCFAs, was identified because calcium citrate was used in comparison with the carbonate form [33]. A lower proportion of *Bacteroidota* bacteria associated with carbohydrate and glycan metabolism in the intestine of agricultural poultry was also registered [34]. Several studies have shown that a high abundance of *Bacillota* in the gut microbiome of broiler chickens is positively correlated with feed efficiency and productivity indicators [35]. Increased Firmicutes have been shown to be positively correlated with energy and nutrient absorption, whereas increased *Bacteroidota* have been associated with poor nutrient absorption [36]. Microbiomes dominated by *Bacteroidota* are characterized by a poorer biodiversity, which will potentially negatively affect productivity [37].

### *Bacillota*/*Bacteroidota* ratio and digestibility

A change in the *Bacillota*/*Bacteroidota* ratio was observed when calcium citrate was used in the diet, which could be one of the reasons for the differences in the digestibility coefficients of feed components between the experimental groups. The relationship between dysbacteriosis development and the *Bacillota*/*Bacteroidota* ratio in the intestinal microbiota is described. Previous studies [36, 38] showed that an increase in the relative abundance of *Bacillota* and the *Bacillota*/*Bacteroidota* ratio in the intestinal microbiota of laying hens has a beneficial effect on the absorption of substances and improves egg production and live weight. However, research data are quite ambiguous and contradictory in describing the influence of the *Bacillota*/*Bacteroidota* ratio on animal productivity and intestinal health [39, 40].

### Changes in SCFA–producing taxa

A more detailed analysis of the taxonomic profile of the cecal microbiota of laying hens fed a diet containing calcium citrate showed a change in the proportion of bacteria (*Oscillospiraceae*, *Lachnospiraceae*, *Lactobacillaceae*), which are the main producers of SCFAs (acetate, butyrate, propionate) and are of fundamental importance for the health of the intestine of vertebrates [41–45]. An increase in the proportion of bacteria of the *Oscillospiraceae* and *Lactobacillaceae* families was detected.

### Functional role of *Oscillospiraceae*

Bacteria of the *Oscillospiraceae* family are carbohydrate degraders that play an important role in maintaining the structure and function of intestinal microbial communities. In a study on broiler chickens, a high abundance of *Oscillospiraceae* bacteria in the intestinal microbiota was positively correlated with FE [46]. Similarly, a positive correlation was noted between the *Oscillospiraceae* taxon and the conversion rates of nitrogen-free substances (r = 0.585) in the experimental group.

### Functional role of *Lactobacillaceae*

Bacteria belonging to the *Lactobacillaceae* family have proteolytic activity, metabolize carbohydrates, and have a positive effect on intestinal health [47]. Lactobacillus bacteria secrete several metabolites that inhibit the growth of opportunistic microflora in the intestines [48]. Increasing the proportion of Lactobacillus bacteria in the cecum of laying hens may have important implications for both fermentation processes and overall bird health as a result of replacing calcium carbonate with the citrate form in the diet [49]. Wen *et al*. [14] reported that the proportion of *Lactobacillaceae* bacteria was significantly higher in hens with higher feed efficiency. Cui Y *et al*. [50] noted that the use of Lactobacillus bacteria in broiler feed improves growth performance and nutrient absorption. A positive relationship was noted between the *Lactobacillaceae* taxon in the cecal microbiota and the conversion rates of crude fat (r = 0.686) and crude protein (r = 0.641). Studies on broiler chickens have shown a positive effect of probiotics from the Bacilli class on the activity of the lipase enzyme, which has a positive effect on the breakdown of fat and fermentation of amino acids [51, 52]. A positive correlation was also noted between the *Lactobacillaceae* taxon and acetic acid levels in the cecum of laying hens. Forte *et al*. [53] observed the same relationship in their study. This study described a positive relationship between the proportion of Lactobacillus bacteria in the ileum of broiler chickens and the release of phosphorus from phytate complexes [13]. This is consistent with our data, where a close correlation was observed between Lactobacillus and phosphorus conversion coefficient (r = 0.588).

### Implications for egg microbiology and vertical transfer

Given the established antibacterial properties of probiotic bacteria and their beneficial impact on gut health, increasing the proportion of Lactobacillus in the cecum could also positively influence egg microbiology. The microbial profile of the cecum of laying hens at the beginning of egg production closely correlates with the composition of the chicken oviduct and intestine microbiome [54, 55]. This indicates a probable transfer of microbiota from hen to chick at the egg formation stage, which will form the basis for the initial intestinal biocenosis of the chick [56].

### Changes in *Lachnospiraceae* and butyrate dynamics

In contrast, a decrease in the proportion of bacteria of the *Lachnospiraceae* family, which are also key producers of butyrate in the cecum of poultry and play an important role in intestinal health, was noted [57, 58]. Butyrate is considered an important metabolite of bacterial origin that supports intestinal energy metabolism. However, we did not observe a decrease in the concentration of SCFAs, including butyric acid, in the cecum. This is probably due to an increase in the proportion of bacteria in the *Oscillospiraceae* and *Lactobacillaceae* families. Bacteria of the *Oscillospiraceae* taxon, as well as *Lachnospiraceae*, are able to use glycans and perform the breakdown and absorption of polysaccharides in chickens with the formation of butyrate [59]. However, the increase in butyric acid concentration in the cecum of laying hens using calcium citrate in the diet was less pronounced than the pronounced increase in acetic and propionic acid content. We assume that this could be a consequence of a decrease in the proportion of bacteria of the *Lachnospiraceae* family in the microbiota of the cecum of the experimental group compared to the control. Nevertheless, we did not find significant correlations between the conversion factors of nutrients and bacteria of the *Lachnospiraceae* family in the microbiota of the cecum of laying hens in the experimental group. However, a negative correlation between the *Lachnospiraceae* taxon in the cecum and the efficiency of feed use in an experiment on chickens has been reported [60].

### Reduction of *Alistipes* and *Fusobacterium*

Among the significant changes in the cecal microbiota of laying hens using calcium citrate in the diet, the proportion of bacteria of the genus *Alistipes* and genus *Fusobacterium* decreased compared with the control. Although these genera of bacteria are commensal microorganisms of the intestine of agricultural poultry that break down carbohydrates, a decrease in their proportion in the microbiota can be considered a positive effect [61]. Data on the role of bacteria of the genus *Alistipes* and genus *Fusobacterium* in metabolic pathways in the intestine and the impact on intestinal health of agricultural poultry are limited. However, studies of the human gastrointestinal microbiota have shown that high levels of *Alistipes* and *Fusobacterium* are correlated with inflammatory bowel disease [62, 63]. Our study revealed a negative correlation between the fiber conversion coefficient and the genus Alistipes. The bacteria of the genus *Fusobacterium* in the gastrointestinal tract of chickens negatively correlated with the body weight of laying hens. The body weight of laying hens in the pre-laying period is the main factor that affects their productivity.

### Mechanistic interpretation of calcium citrate action

The enrichment of *Lactobacillaceae* and *Oscillospiraceae* with the simultaneous reduction of *Bacteroidota* suggests a metabolic shift toward energy metabolism. This provides new evidence linking the specific form of dietary calcium to gut microbiome functionality.

We propose that the mechanism by which calcium citrate acts on the cecal microbiota of laying hens, specifically on lactic acid bacteria, stems from its dual composition. Unlike other calcium sources, the citrate form delivers both the essential mineral and an organic acid, which likely leads to an improved microbial ecology. Calcium regulates the activity and stabilizes the structure of several bacterial enzymes, such as α-amylase and β-galactosidase. We hypothesize that the greater amount of calcium available in the intestine enhances this enzymatic activity, thereby facilitating the active proliferation of these beneficial bacteria [64, 65]. A positive relationship has been noted between calcium and the proportion of bacteria in the intestine that have probiotic properties [66, 67]. Our study revealed a positive correlation between the *Lactobacillaceae* taxon and calcium conversion coefficient (r = 0.698).

It should also be noted that changes in the microbiota of the cecum could be due not only to an increase in available calcium but also to the presence of citrate, an organic acid in the preparation. A positive effect of citrate metabolism on lactic acid bacteria growth has been described [12, 68]. Lactic acid bacteria actively metabolize citrate, producing a range of specific enzymes, including permease, citrate lyase, and oxaloacetate decarboxylase. These enzymes break down citrate into pyruvate, acetate, and other metabolites, a process linked to adenosine triphosphate production. The resulting metabolites serve as vital energy sources for bacteria and help them maintain a stable intracellular pH. This mechanism favorably affects both bacterial growth and resilience to acid stress [69]. Citric acid also helps to reduce the number of pathogens in the cecum, including *Alistipes* bacteria, which are associated with intestinal inflammation [70, 71].

### Functional pathway modulation and metabolomic relevance

Along with the change in the taxonomic composition of the cecum of laying hens, the KEGG function profiles also differed between groups I and II. Analysis of the predicted metabolic pathways of the cecal microbiota showed a positive trend in the abundance of genes of some carbohydrate metabolism pathways (starch and sucrose metabolism, pyruvate metabolism) in the experimental group compared with the control, which is consistent with the data on the increase in the digestibility of nitrogen-free extractives in our study. Pyruvate metabolism is closely associated with butyrate production and bacteria of the Ruminococcaceae family in the cecum of laying hens [72, 73]. Our study noted an increase in the concentration of butyric acid and the proportion of bacteria of the Ruminococcaceae family in the cecum of laying hens in the experimental group compared with the control group. We also found a positive correlation (r = 0.544) between butyric acid concentration in the cecum and bacteria of the Ruminococcaceae family.

A positive trend in gene abundance was also noted for metabolic pathways related to amino acid metabolism in the experimental group. In a study by Huang *et al*. [74], enrichment of genes in bacterial metabolic pathways in the gut related to starch and sugar metabolism, as well as phenylalanine, tyrosine, and tryptophan biosynthesis, was noted in broilers with high feed efficiency. Similarly, an increase in the digestibility coefficient of crude protein was noted when calcium carbonate was replaced by calcium citrate in the diet. We assume that an increase in the abundance of genes associated with amino acid metabolism will positively affect egg quality. Egg protein contains almost 50% phenylalanine, tyrosine, and tryptophan. Obianwuna *et al*. [75] described a positive relationship between improved nutrient digestibility, particularly amino acids, and egg protein quality against the background of using probiotics in laying hen feed.

Functional profiling of the cecum microbiota showed that the treated group had a higher proportion of lipid metabolism genes (fatty acid breakdown and biosynthesis) than the control group. Chen *et al*. [76] found that lipid metabolism pathways, including fatty acid biosynthesis and fatty acid degradation, were found in higher abundance in chickens with low abdominal fat deposition. A study by Cui *et al*. [77] showed that lower abdominal fat in laying hens was associated with a reduced proportion of Rikenellaceae bacteria and an increased concentration of butyrate in the intestine. Similarly, in our study, the experimental group had a lower proportion of bacteria of the Rikenellaceae family, an increased concentration of butyrate in the cecum, and a greater abundance of fatty acid metabolism genes than the control group, which we assume will have a positive effect on the health and lipid metabolism of laying hens.

### Novelty, sustainability, and concluding synthesis

To the best of our knowledge, this is the first study to demonstrate that substituting inorganic calcium carbonate with calcium citrate alters the functional pathways of the cecal microbiota associated with amino acid and fatty acid metabolism in laying hens. The integration of 16S rRNA sequencing with KEGG pathway prediction elevates the functional interpretation of mineral nutrition in poultry, distinguishing this work from traditional digestibility studies and contributing to an emerging research field.

Changes in the taxonomic profile and functioning of the cecum microbiota of laying hens with calcium source replaced in the experimental group were naturally associated with the dynamics of microbial SCFAs in the intestine, which affected feeding efficiency. This could be the main reason for the higher absorption of calcium and phosphorus in the body of laying hens in the experimental group compared with the control. Acetic, propionic, and butyric acids can affect the absorption of macro- and microelements in the intestine. The mechanism of the effect of SCFAs on the skeletal system has been extensively studied [78].

**Figure 11:**
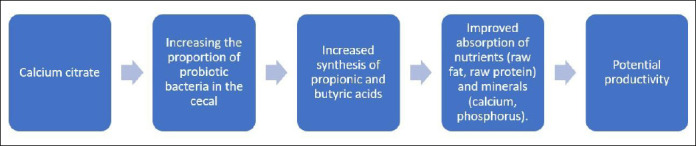
Mechanistic model reflecting the effect of calcium citrate on intestinal microbiota and nutrient absorption in laying hens.

In summary, replacing the dietary calcium source triggered a cascade of changes in nutrient absorption and gut microbiota composition in laying hens. We hypothesize that these changes will positively impact subsequent productivity (Figure 11). Our study integrated nutrition, microbiology, and metabolomics to examine nutrient absorption and gut microbiota function during the critical pre-laying period. This interdisciplinary approach enhances understanding of the diet–microbiome–metabolism axis in poultry and contributes to the growing field of nutri-microbiome science.

### Sustainability and One Health implications

The findings align with FAO objectives for sustainable livestock systems and SDG 12. Substitution of calcium carbonate with calcium citrate increased crude protein (p ≤ 0.001) and crude fat digestibility (p ≤ 0.001), reducing feed requirements per unit egg mass and lowering environmental pressure. Improved nutrient digestibility also reduces nitrogen and phosphorus excretion, mitigating eutrophication risks and supporting SDG 12.2 and 12.4.

Calcium citrate offers a dual nutritional and microbiota-modulating strategy to improve gut health, enhance feed conversion, reduce antibiotic reliance, and support One Health and Farm to Fork strategies. Finally, chemical synthesis of calcium citrate from dolomite flour represents an environmentally sustainable approach that reduces mining waste while providing a highly digestible calcium source for poultry production.

## CONCLUSION

This study demonstrated that replacing calcium carbonate with calcium citrate during the pre-laying period significantly improved nutrient utilization and gut microbiota functionality in laying hens. The experimental group showed markedly higher digestibility coefficients for dry matter, organic matter, crude protein, crude fat, Ca, and P, accompanied by increased concentrations of cecal SCFA, particularly acetic, propionic, and butyric acids. Microbiota analysis revealed increased richness and diversity, enrichment of *Bacillota*, especially *Lactobacillaceae* and *Oscillospiraceae*, and a reduced abundance of *Bacteroidota*, including *Alistipes* and *Fusobacterium*. Functional prediction based on 16S rRNA sequencing indicated enrichment of microbial pathways related to carbohydrate, amino acid, and lipid metabolism, alongside reduced methane metabolism.

From a production perspective, improved digestibility of protein, fat, Ca, and P directly translates into enhanced FE and reduced nutrient excretion, supporting both economic efficiency and environmental sustainability. The modulation of cecal microbiota toward SCFA-producing and probiotic-associated taxa suggests additional benefits for gut health, mineral absorption, and metabolic efficiency. These outcomes are particularly relevant during the pre-laying period, when intestinal and metabolic programming critically influence subsequent egg production, eggshell quality, and skeletal integrity.

Key strengths include the focus on the underexplored pre-laying period, the integration of physiological balance trials with microbiota profiling, and the combined taxonomic and functional interpretation of gut microbial changes using 16S rRNA sequencing and KEGG-based prediction. This integrative approach provides mechanistic insight into how calcium source influences nutrient absorption through microbiota-mediated pathways, moving beyond conventional digestibility studies.

The study was conducted using a single commercial cross and a limited number of samples for microbiota analysis, which may restrict broader generalization. Additionally, functional predictions were inferred from marker-gene sequencing rather than confirmed by metatranscriptomic or metabolomic analyses.

Future research should validate these findings across different genetic lines and production systems, extend observations into the laying phase to assess impacts on egg production and eggshell quality, and incorporate multi-omics approaches to directly quantify microbial metabolic activity. Long-term studies evaluating bone strength, mineral retention, and lifetime productivity are also warranted.

Overall, dietary substitution of calcium carbonate with calcium citrate during the pre-laying period induces favorable shifts in nutrient digestibility, cecal microbiota composition, and microbial metabolic potential. These coordinated changes likely underpin the improved Ca and P utilization observed and may confer lasting benefits for productivity and health in laying hens. The findings support calcium citrate as a promising nutritional strategy within an integrated nutri-microbiome framework for sustainable poultry production.

## DATA AVAILABILITY

Raw Illumina reads were submitted to the National Center for Biotechnology Information SRA under BioProject accession number SUB15800169 (https://submit.ncbi.nlm.nih.gov/subs/biosample/SUB15800169/ overview). The data supporting the findings of this study are available upon reasonable request from the corresponding author.

## AUTHORS’ CONTRIBUTIONS

ES and TKh: Conceptualization, methodology, and data curation. DS and AK: Validation and formal analysis. EY, DS, and KR: Investigation and collection of samples. TK, AM, KR, and KN: Validation, format analysis, and drafted the manuscript. EY, ES, and TKh: writing, review, and editing. All authors have read and approved the final version of the manuscript.
